# A study on the correlation of placental anastomosis and superficial vascular branches of selective fetal growth restriction in monochorionic diamniotic twins

**DOI:** 10.1186/s12884-023-06157-5

**Published:** 2023-11-30

**Authors:** Zhiman Lin, Xueju Wang, Luyao Li, Pengbo Yuan, Yangyu Zhao, Yuan Wei

**Affiliations:** Department of Obstetrics and Gynecology, Peking University Third Hospital, No.49 Hua Yuan North Road, Hai Dian District, Beijing, 100191 China

**Keywords:** Selective fetal growth restriction, Vascular anastomosis, Placental superficial vascular branch, Ultrasound, Umbilical vessel Doppler, Monochorionic twin placentas

## Abstract

**Introduction:**

The main purpose of the present study was to investigate the correlation between placental anastomosis and superficial vascular branches in selective fetal growth restriction (sFGR) in monochorionic diamniotic twins.

**Materials and methods:**

This was a retrospective analysis of the pregnancy data and placental perfusion of 395 patients with monochorionic diamniotic (MCDA) twin pregnancies delivered at our hospital from April 2013 to April 2020. We divided the patients into two groups and compared the number of placental superficial vascular branches in sFGR twins and normal MCDA twins. The correlation between the placental anastomosis and the number of superficial vascular branches in sFGR and normal MCDA twins was also investigated.

**Results:**

The number of umbilical arterial branches and umbilical venous branches was less than larger twins in sFGR, larger twins in normal MCDA and smaller twins in normal MCDA. (11.83 [4–44], 21.82 [7–50], 19.72 [3–38], 14.85 [0–31], *p* < 0.001, 6.08 [1–18], 9.60 [3–22], 9.96 [2–22], 8.38 [1–20], *p* < 0.00) For smaller twins in the sFGR group, the number of umbilical venous branches was positively associated with AA anastomosis overall diameter, AV anastomosis overall diameter and all anastomosis overall diameter. (*r* = 0.194, 0.182 and 0.211, *p* < 0.05)

**Conclusions:**

The risk of sFGR may arise when the placenta from MCDA twins shows a poor branching condition of placental superficial vessels. For the smaller twin of sFGR, regular ultrasound examination of the number of the umbilical venous branches may help to predict artery-to-artery (AA) overall diameter, artery‐to‐vein (AV) overall diameter and all anastomosis overall diameter.

## Key message

The risk of sFGR may arise when the placenta from MCDA twins shows a poor branching condition of placental superficial vessels. For the smaller twin of sFGR, regular ultrasound examination of the number of the umbilical venous branches may help to predict artery-to-artery (AA) overall diameter, artery-to-vein (AV) overall diameter and all anastomosis overall diameter.

## Introduction

Selective fetal growth restriction (sFGR), which affects approximately 10–15% of monochorionic diamniotic (MCDA) twin pregnancies [1], is a complication unique to MCDA twin pregnancies. It is generally accepted that the primary etiology of sFGR is based on unequally distributed placental sharing, abnormal umbilical cord insertion and a distinct placental superficial vascular structure [2, 3].

The placental superficial vessels, which are continuous with the umbilical arteries and veins, begin to branch at the cord insertion site. There are then superficial vascular anastomoses that form between two pairs and communicate the two circulations in MCDA twin placentas. Recent evidence has shown that type of the umbilical cord insertion in MCDA twins, which may lead to the presence of sFGR, correlates with the placental superficial vessels. De Paepe et al. [4] reported a study of 138 MCDA placentas and found that co-twin placentas with velamentous cord insertion had a significantly higher distribution of placental superficial vessels than those with paracentral cord insertion. Considering the critical importance of the non-central cord in the pathophysiology of sFGR, the placental superficial vessels may also be involved in the presence of sFGR. Furthermore, previous studies have shown that the progression and prognosis of sFGR may be influenced by transfusion imbalances between the twins due to placental superficial vascular anastomoses [2]. In view of the above, the placental superficial vessels of sFGR may be related to the vascular anastomosis. However, little is known about the relationship between the placental superficial vessels and vascular anastomosis in sFGR of MCDA twin pregnancies. As a result, the relationship between the placental anastomosis and superficial vascular branches of sFGR has not yet been determined. The aim of this study was to explore the characteristics of the placental superficial vascular branches of sFGR. We also looked at how placental anastomosis characteristics correlated with superficial vascular branches in sFGR.

## Materials and methods

To perform the case-control study, all consecutive cases of MCDA pregnancies admitted to the Department of Obstetrics in Peking University Third Hospital from April 2013 to April 2020 were enrolled. Cases of MCDA pregnancies detected twin-to- twin transfusion syndrome (TTTS) or twin anemia-polycythemia sequence (TAPS) concurrently, were disregarded. We collected every placenta after obtaining informed consent from the patient. Cases in which the placenta was damaged following delivery were also excluded.

Routine ultrasound scans were performed every two weeks in patients with MCDA twins. We mainly focused on four indicators, abdominal circumference (AC), estimated fetal weight (EFW), EFW discordance and umbilical arterial pulsatility index to determine if sFGR was present. The diagnosis of sFGR in MCDA twin pregnancies was confirmed when the EFW of either twin was less than the 3rd centile corresponding gestation age [1]. As long as two out of four parameters (AC of either twin < 10th centile, EFW of either twin < 10th centile, EFW discordance ≥ 25%, UA-PI of the smaller twin > 95th centile) were met, the diagnosis of sFGR in MCDA twin pregnancies was also established [1–3]. TTTS was diagnosed as oligohydramnios in one twin (maximum vertical pocket (MVP) ≤ 2 cm) and polyhydramnios in the other (MVP ≥ 8 cm before 20 weeks and MVP ≥ 10 cm after 20 weeks) [5]. Prenatal diagnosis of TAPS was based on combined presence of the middle cerebral artery peak systolic velocity (MCA-PSV) being greater than 1.5 the multiples of the median [MoM] in anaemic donors and less than 1.0 MoM in polycythemic recipients [6]. Prenatal diagnosis of TAPS was established when the differences in inter-twin was larger than 8 g/dL [6]. Patients in the remainder of the cases were divided into two groups on the basis of whether or not they had a diagnosis of sFGR.

We collected general characteristics of patients included age, gestational age at delivery, assisted reproductive technology use and fetal reduction in the first trimester. Gestational age was defined according to last menstrual period and with ultrasound measurement of crown-rump length at the first trimester. Pregnancy-induced hypertension, gestational diabetes mellitus and birthweight of each baby were also recorded.

**Fig. 1:**
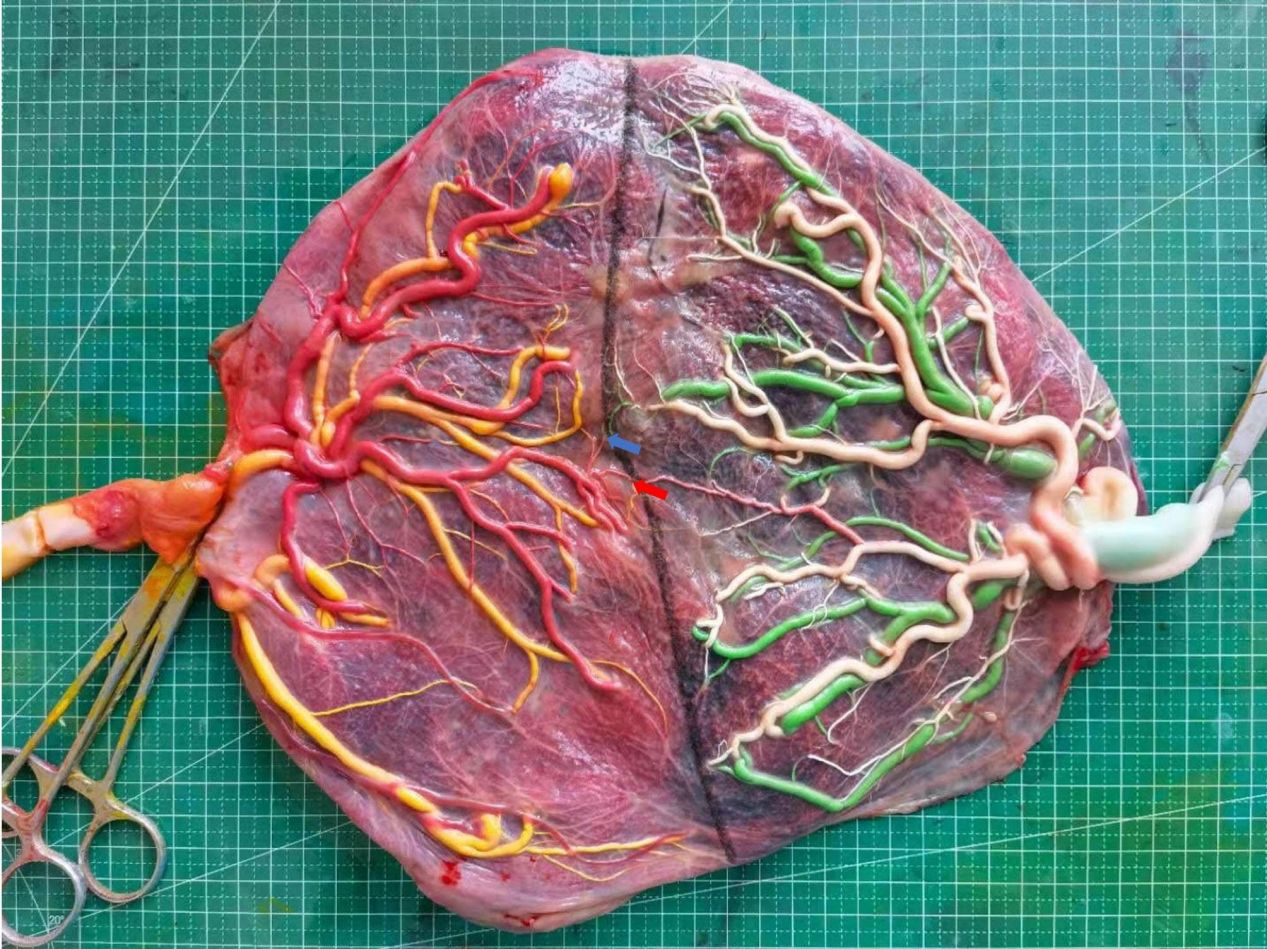
Representative placenta of MCDA twins post dye injection (Red arrow: AA anastomosis; blue arrow: AV anastomosis; black curve: vascular equator)

In order to determine the final chorionic diagnosis, we regularly examined the placentas of patients with MCDA pregnancies at our center. If the chorionic diagnosis of the placenta was still uncertain, a Pathological examination was carried out. All placentas were fully injected with colored dye to clearly manifest the pattern of placental anastomosis and superficial vascular branch. The digital pictures shown in Fig. 1 were saved, and we used ImageJ (v. 1.51j8, NIH, MD, USA) for subsequent analysis. Based on the direct external connection of homonymous pairs of umbilical vessels, superficial artery-artery (AA) anastomosis and vein-vein (VV) anastomosis were defined. Deep artery-vein (AV) anastomosis was defined as the penetration of a single unpair artery from one twin across the chorionic plate within less than 1.0 mm of a single unpair vein from the other one [2]. Measurements of AA diameter and VV diameter were taken in the smallest portion, and AV diameter was taken on the arterial side. Placental anastomosis type and diameter were measured. The number of superficial vascular branches was also measured for this study.

All analyses were performed using SPSS 26.0 statistics (IBM Corp, Armonk, NY, USA). Enumeration data were expressed as *n* (%). Otherwise, the measurement data were expressed as mean ± standard deviation or expressed as median (maximum value, minimum value). The first step was to determine whether the measurement data were normally distributed. To allow further comparison of general characteristics, a t-test was used if all groups had a normal distribution and data were expressed as mean ± standard. If not, data were expressed as medium (maximum, minimum) and analysed using a nonparametric test to detect differences between groups. Spearman’s method was used for the analysis of correlation. Statistical significance was considered to be a two-sided probability value of *p* < 0.05.

All placentas were obtained following approval from the Ethics Committee of Peking University Third Hospital (Number: M2016199, Date: 8 March 2017) and patient informed consent.

## Results

**Fig. 2:**
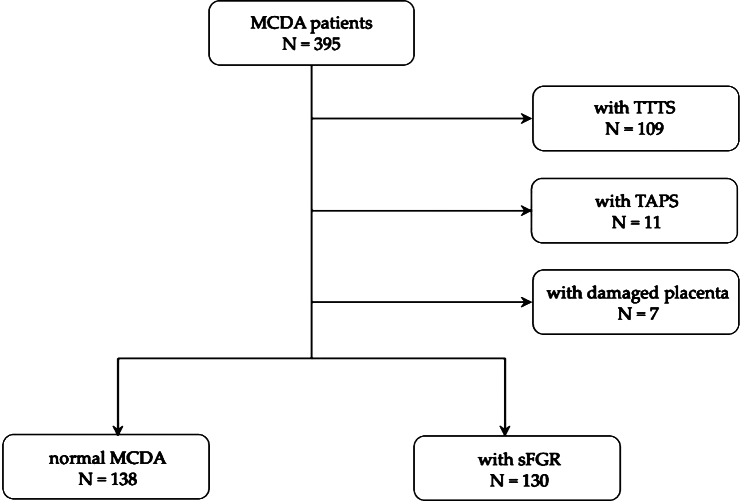
Flowchart of patients included in the study (MCDA: monochorionic diamniotic, sFGR: selective fetal growth restriction, TTTS: twin to twin transfusion syndrome, TAPS: twin anemia polythemia sequence)

In total, 395 patients with MCDA twin pregnancy delivered at the Obstetrics Department of the Peking University Third Hospital between April 2013 and April 2020, were recruited. Furthermore, 109 patients with TTTS, 11 patients with TAPS, and 7 patients with placental damage were unavailable for analysis in this study. The left 268 patients with complete dye injection were separated into two groups: 138 normal MCDA and 130 sFGR. The patient selection procedure for this study is presented in Fig. 2.

Findings on general characteristics are presented in Table 1. In our series, the prevalence of assisted reproductive technology use was significantly lower in sFGR compared to normal MCDA. Patients in the sFGR group also exhibited a significantly lower gestational age at delivery in comparison with those in the normal MCDA group. The birthweights of both larger and smaller twins were both significantly lower in sFGR than in normal MCDA. Meanwhile, the birthweight discordance ratio was significantly higher in sFGR than in normal MCDA. The comparisons of placental superficial vascular branches in the two groups are presented in Table 2, respectively. As we can see, both umbilical arterial branches and umbilical venous branches were significantly less in the smaller twin of sFGR than in the larger twin of sFGR or in both twins of normal MCDA.

**Table 1:** Comparisons of general characteristics associated with cases of sFGR and normal MCDA

	sFGR group *n* = 130	Normal MCDA group *n* = 138	*p*-value
Advanced maternal age, *n* (%)	23(17.7)	27(24.7)	0.928
Assisted reproductive technology use, *n* (%)	12(9.2)	32(23.2)	0.002
Fetal reduction in the first trimester, *n* (%)	2(1.5)	5(3.6)	0.285
Pregnancy induced hypertension, *n* (%)	42(32.3)	37(26.8)	0.324
Gestational diabetes mellitus, *n* (%)	25(19.6)	32(23.2)	0.429
Gestational age at delivery, *week* (range)	33.527(24.0-37.3)	35.182(19.4–38.6)	0.000
Birthweight of larger twins, *g* (range)	2069.59(740–3230)	2405.03(320–3510)	0.000
Birthweight of smaller twins, *g* (range)	1453.23(530–2480)	2192.43(290–3110)	0.000
Birthweight discordance ratio, *n* (range)	0.30(0.04–0.63)	0.10(0.00–1.00)	0.000

Abbreviations: sFGR, selective fetal growth restriction; MCDA, monochorionic diamniotic

**Table 2:** Comparisons of placental superficial vascular branches in cases of sFGR and normal MCDA

	sFGR group		Normal MCDA group		*h*-value		*p*-value		
	larger twin *n* = 130	smaller twin *n* = 130	larger twin *n* = 138	smaller twin *n* = 138					
Umbilical arterial branches, *n* (range)	21.82(7–50)	11.83(4–44)	19.72(3–38)	14.85(0–31)		139.142		0.000	
Umbilical venous branches, *n* (range)	9.60(3–22)	6.08(1–18)	9.96(2–22)	8.38(1–20)		100.178		0.000	
Umbilical vascular overall branches, *n* (range)	31.42(12–65)	17.87(5–52)	29.68(5–55)	23.23(4–42)		155.084		0.000	

Abbreviations: sFGR, selective fetal growth restriction; MCDA, monochorionic diamniotic

The results of the Spearman correlation analysis between placental anastomosis and superficial vascular branches are presented in the Tables 3, 4 and 5. For smaller twins in the sFGR group, the number of umbilical venous branches was positively associated with AA anastomosis overall diameter (*r* = 0.194, *p* = 0.029), and it was also positively.

**Table 3:** Spearman rank correlation coefficients between placental anastomosis and branching numbers of placental superficial vessels in cases of smaller twins of sFGR (*r*, (*p*-value))

	AA overall diameter	AV overall diameter	VV overall diameter	All anastomosis overall diameter	
The number of umbilical arterial branches	0.009(0.919)	0.017(0.848)	-0.007(0.941)	0.052(0.563)	
The number of umbilical venous branches	0.194(0.029)	0.182(0.040)	0.023(0.806)	0.211(0.017)	
The overall number of umbilical vascular branches	0.035(0.695)	0.045(0.615)	0.013(0.886)	0.077(0.386)	

Abbreviations: sFGR, selective fetal growth restriction; AA, artery-to‐artery; AV,

artery‐to‐vein; VV, vein‐to‐vein

**Table 4:** Spearman rank correlation coefficients between placental anastomosis and branching numbers of placental superficial vessels in cases of larger twins of normal MCDA (*r*, (*p*-value))

	AA overall diameter	AV overall diameter	VV overall diameter	All anastomosis overall diameter	
The number of umbilical arterial branches	0.058(0.496)	0.169(0.048)	-0.043(0.671)	0.108(0.205)	
The number of umbilical venous branches	0.026(0.761)	0.195(0.022)	0.146(0.152)	0.156(0.067)	
The overall number of umbilical vascular branches	0.053(0.535)	0.205(0.016)	0.021(0.836)	0.139(0.104)	

Abbreviations: MCDA, monochorionic diamniotic; AA, artery-to‐artery; AV,

artery‐to‐vein; VV, vein‐to‐vein

**Table 5:** Spearman rank correlation coefficients between placental anastomosis and branching numbers of placental superficial vessels in cases of smaller twins of normal MCDA (*r*, (*p*-value))

	AA overall diameter	AV overall diameter	VV overall diameter	All anastomosis overall diameter	
The number of umbilical arterial branches	0.097(0.258)	0.168(0.049)	-0.084(0.408)	0.116(0.174)	
The number of umbilical venous branches	-0.030(0.723)	0.222(0.009)	-0.063(0.537)	0.093(0.278)	
The overall number of umbilical vascular branches	0.043(0.616)	0.230(0.007)	-0.061(0.550)	0.134(0.117)	

Abbreviations: MCDA, monochorionic diamniotic; AA, artery-to‐artery; AV, artery‐to‐vein; VV, vein‐to‐vein

associated with all anastomosis overall diameter (*r* = 0.211, *P* = 0.040). AV anastomosis overall diameter of larger twins in the normal MCDA group was positively correlated with the number of umbilical arterial branches (*r* = 0.169, *P* = 0.048). It was also positively correlated with the overall number of umbilical vascular branches (*r* = 0.205, *P* = 0.016). AV anastomosis overall diameter of smaller twins in the normal MCDA group was positively correlated with the number of umbilical arterial branches (*r* = 0.230, *P* = 0.007). It was also positively correlated with the overall number of umbilical vascular branches (*r* = 0.230, *P* = 0.007). For smaller twins in the sFGR group, larger twins in the normal MCDA group and smaller twins in the normal MCDA group, the number of umbilical venous branches was positively correlated with AV anastomosis overall diameter (*r* = 0.182, 0. 195 and 0.222; *P* = 0.040, 0.022 and 0.009, respectively). No correlation was found between the other indicators.

## Discussion

Our study showed that twins with sFGR had significantly different branching conditions of the placental superficial vessels as compared with twins with normal MCDA. More specifically, the smaller twin in the sFGR group had fewer umbilical arterial branches and umbilical venous branches than the other twin in the two groups. Otherwise, we demonstrated that the number of umbilical venous branches was positively correlated with the AA anastomosis overall diameter, the AV anastomosis overall diameter, and all anastomosis overall diameter in smaller twins in the sFGR group. These results mean that in smaller twins in the sFGR group, the smaller the number of umbilical venous branches, the smaller the AA anastomosis overall diameter, the AV anastomosis overall diameter, and all anastomosis overall diameter. Some ideas about the aetiology and prediction of sFGR seem to emerge from the above findings. Velamentous cord insertion, which is more common in twins than in singletons [7], has been associated with reduced placental superficial vessels in singletons [8, 9]. Similar to singletons, there is a strong correlation between the cord insertion type and the placental superficial vessels in MCDA twins [4]. In a study of 138 MCDA placentas, De Paepe et al. [4] reported that the part of the placenta with velamentous cord insertion showed a significantly higher superficial vascular distribution than the other part with paracentral cord insertion. Velamentous cord insertion may be the cause of unequal nutrient supply to the twins in MCDA [7, 10]. The presence of sFGR, which is thought to be associated with the non-central cord insertion in at least one twin of MCDA [11, 12], may also be associated with placental superficial vessels. However, there is a lacking of previous studies investigating the association between placental superficial vascular branches and the presence of FGR.

In the present study, we demonstrated that the smaller twin in the sFGR group had fewer umbilical arterial branches and umbilical venous branches than the other twin in the two groups, which indicated that the branching angiogenesis of placental superficial vessels may differ between these groups. The poor branching condition of placental superficial vessels may reduce transport efficiency. In summary, the poor branching condition of placental superficial vessels may be implicated in the mechanism of development and progression of sFGR. However, there is a lacking of previous studies investigating the association between placental superficial vascular branches and the presence of FGR. In the present study, we demonstrated that the placental superficial vascular branches differed significantly between twins of normal MCDA and sFGR, indicating that placental superficial branching angiogenesis may differ between these groups. It is possible that poor placental superficial vascular branching plays a role in the mechanism of development and progression of sFGR.

The distinctive placental anastomosis, which contributes to adverse outcomes in MCDA twins, leads to a shared circulation between twins [10]. Types of placental anastomoses include AA, AV and VV [10, 13]. The bidirectional blood flow in the AA and VV anastomosis can be regulated based on the hemodynamic pressure between the two pairs. In contrast, deep AV anastomosis has a unidirectional blood flow and leads to transfusion imbalance. These anastomoses allow twins in MCDA to exchange blood with each other [10, 13–15].

The number of umbilical venous branches in the smaller twin in the sFGR group was positively correlated with the AA anastomosis overall diameter, the AV anastomosis overall diameter and all anastomosis overall diameter according to the present study. These results showed that the fewer the umbilical venous branches in the smaller twin in the sFGR group, the smaller the AA anastomosis overall diameter and all anastomosis overall diameter. In theory, a smaller overall anastomosis diameter is associated with less blood exchange [10, 14], which implies fewer blood nutrients and oxygen compensation from the larger twin to the smaller one leading to a worse outcome of sFGR [14]. Furthermore, the Doppler ultrasound techniques can be used to measure the number of umbilical vascular branches [16]. Hence, for the smaller twin in sFGR, regular ultrasound examinations of the number of umbilical venous branches can be a predictor to estimate the AA anastomosis overall diameter, the AV anastomosis overall diameter and all anastomosis overall diameter.

The limitations of the present study have to be mentioned. There is still a great deal of uncertainty about the exact mechanistic basis of these placental vascular phenomena, and further studies are required. Moreover, although the present study showed some statistically significant correlations between placental anastomosis and superficial vascular branches in cases of smaller twins in the sFGR group, the correlations (Spearman’s rank) are quite weak. This means that the conclusion needed to be interpreted with caution. Hopefully, this preliminary result will provide a basis for future research.

## Conclusions

In conclusion, the results of this study suggest that the poor branching condition of placental superficial vessels in MCDA may lead to the presence of sFGR. For the 1smaller twin in sFGR, routine ultrasound examination of the number of umbilical venous branches may help to predict the AA anastomosis overall diameter, the AV anastomosis overall diameter and all anastomosis overall diameter.

## Data Availability

The datasets during the current study are available from the corresponding author on reasonable request.

## List of abbreviations

MCDA: Monochorionic diamniotic
sFGR: Selective fetal growth restriction
AA: Artery-to-artery
AV: Artery-to-vein
VV: Vein-to-vein

## References

[1] Khalil A, Beune I, Hecher K, Wynia K, Ganzevoort W, Reed K, Lewi L, Oepkes D, Gratacos E, Thilaganathan B, Gordijn SJ (2019). Consensus definition and essential reporting parameters of selective fetal growth restriction in twin pregnancy: a Delphi procedure. Ultrasound Obstet Gynecol.

[2] Zhao DP, de Villiers SF, Slaghekke F, Walther FJ, Middeldorp JM, Oepkes D, Lopriore E (2013). Prevalence, size, number and localization of vascular anastomoses in monochorionic placentas. Placenta.

[3] Buca D, Pagani G, Rizzo G, Familiari A, Flacco ME, Manzoli L, Liberati M, Fanfani F, Scambia G, D’Antonio F (2017). Outcome of monochorionic twin pregnancy with selective intrauterine growth restriction according to umbilical artery doppler flow pattern of smaller twin: systematic review and meta-analysis. Ultrasound Obstet Gynecol.

[4] De Paepe ME, Shapiro S, Hanley LC, Chu S, Luks FI (2011). Correlation between cord insertion type and superficial choriovasculature in diamniotic-monochorionic twin placentas. Placenta.

[5] Quintero RA, Dickinson JE, Morales WJ, Bornick PW, Bermudez C, Cincotta R, Chan FY, Allen MH (2003). Stage-based treatment of twin-twin transfusion syndrome. Am J Obstet Gynecol.

[6] Baschat AA, Miller JL (2022). Pathophysiology, diagnosis, and management of twin anemia polycythemia sequence in monochorionic multiple gestations. Best Pract Res Clin Obstet Gynaecol.

[7] Costa-Castro T, Zhao DP, Lipa M, Haak MC, Oepkes D, Severo M, Montenegro N, Matias A, Lopriore E. Velamentous cord insertion in dichorionic and monochorionic twin pregnancies - does it make a difference? Placenta 2016, 42, 87–92, 10.1016/j.placenta.2016.04.007. 10.1016/j.placenta.2016.04.007 27238718

[8] Salafia CM, Shah RG, Misra DP, Straughen JK, Roberts DJ, Troxler L, Morgan SP, Eucker B, Thorp JM (2017). Chorionic vascular fit in the human placenta: relationship to fetoplacental outcomes. Placenta.

[9] Yampolsky M, Salafia CM, Shlakhter O, Haas D, Eucker B, Thorp J. Centrality of the umbilical cord insertion in a human placenta influences the placental efficiency. Placenta 2009, 30, 1058–64, 10.1016/j.placenta.2009.10.001. 10.1016/j.placenta.2009.10.001 PMC2790011 19879649

[10] Lewi L, Deprest J (2013). Hecher,K.The vascular anastomoses in monochorionic twin pregnancies and their clinical consequences. Am J Obstet Gynecol.

[11] De Paepe ME, Shapiro S, Young L, Luks FI (2010). Placental characteristics of selective birth weight discordance in diamniotic-monochorionic twin gestations. Placenta.

[12] Lin D, Fan D, Wu S, Rao J, Zhang H, Chen T, Liu J, Ye S, Zeng M, Liu Y (2020). Role of velamentous cord insertion in monochorionic twin pregnancies: a PRISMA-compliant systematic review and meta-analysis of observational studies. J Matern Fetal Neonatal Med.

[13] Lopriore E, Slaghekke F, Middeldorp JM, Klumper FJ, van Lith JM, Walther FJ, Oepkes D. Accurate and simple evaluation of vascular anastomoses in monochorionic placenta using colored dye. J Vis Exp. 2011;(55):e3208. 10.3791/3208. 10.3791/3208 PMC3230184 21912373

[14] Nikkels PG, Hack KE, van Gemert MJ (2008). Pathology of twin placentas with special attention to monochorionic twin placentas. J Clin Pathol 2008.

[15] Kalafat E, Thilaganathan B, Papageorghiou A, Bhide A, Khalil A (2018). Significance of placental cord insertion site in twin pregnancy. Ultrasound Obstet Gynecol.

[16] Hernandez-Andrade E, Huntley ES, Bartal MF, Soto-Torres EE, Jaiman S, Johnson A (2022). Doppler evaluation of normal and abnormal placenta. Ultrasound Obstet Gynecol.

